# The Ankyrin Repeat Domain of the TRPA Protein Painless Is Important for Thermal Nociception but Not Mechanical Nociception

**DOI:** 10.1371/journal.pone.0030090

**Published:** 2012-01-25

**Authors:** Richard Y. Hwang, Nancy A. Stearns, W. Daniel Tracey

**Affiliations:** 1 Department of Anesthesiology, Duke University Medical Center, Durham, North Carolina, United States of America; 2 Department of Cell Biology, Duke University Medical Center, Durham, North Carolina, United States of America; 3 Department of Neurobiology, Duke University Medical Center, Durham, North Carolina, United States of America; Yale School of Medicine, United States of America

## Abstract

The *Drosophila* TRPA channel Painless is required for the function of polymodal nociceptors which detect noxious heat and noxious mechanical stimuli. These functions of Painless are reminiscent of mammalian TRPA channels that have also been implicated in thermal and mechanical nociception. A popular hypothesis to explain the mechanosensory functions of certain TRP channels proposes that a string of ankyrin repeats at the amino termini of these channels acts as an intracellular spring that senses force. Here, we describe the identification of two previously unknown Painless protein isoforms which have fewer ankyrin repeats than the canonical Painless protein. We show that one of these Painless isoforms, that essentially lacks ankyrin repeats, is sufficient to rescue mechanical nociception phenotypes of *painless* mutant animals but does not rescue thermal nociception phenotypes. In contrast, canonical Painless, which contains Ankyrin repeats, is sufficient to largely rescue thermal nociception but is not capable of rescuing mechanical nociception. Thus, we propose that in the case of Painless, ankryin repeats are important for thermal nociception but not for mechanical nociception.

## Introduction

Transient receptor potential (TRP) channels are important in the senses of vision, taste, touch, hearing, nociception (mechanical, thermal, and chemical), and thermosensation (reviewed in [1]). *painless* encodes a *Drosophila* TRPA channel that is required for both thermal and mechanical nociception in *Drosophila* [2]–[5]. In addition, gustatory avoidance of isothiocyanate compounds, and gravity perception in adult flies require *painless*. The molecular mechanisms that allow *painless* to have polymodal sensory roles that include thermosensory, chemosensory, and mechanical signaling are not yet understood.

The nociception function of *painless* was initially found through the investigation of nocifensive escape locomotion (NEL) behavior that is seen in larvae exposed to noxious heat [4]. When performing NEL, larvae rotate around the anterior posterior axis in a corkscrew-like manner. *painless* mutant larvae show increased sensory thresholds for nocifensive responses to noxious heat as well as responses to noxious mechanical stimuli. Evidence suggests that the Painless channel is a direct sensor of noxious heat. For example, electrophysiological recordings from *painless* mutant larval abdominal thermosensory neurons showed decreased firing in response to noxious temperatures [4] and studies in heterologous expression systems have shown that Painless is a heat activated thermoTRP with a threshold of approximately 39–42°C [3]. This *in vitro* heat activation threshold for Painless is similar to the 39–41°C behavioral threshold for triggering larval NEL. The nociceptive function for Painless is likely mediated by nociceptive Class IV multidendritic (mdIV) neurons which express the gene and also show strong increases in firing at temperatures >39–41°C [6]. Evidence suggests that mdIV neurons are nociceptors since optogenetic activation of the mdIV neurons is sufficient to trigger NEL and blocking the synaptic output of the mdIV neurons shows that these neurons are necessary for responses to heat and mechanical stimulation [7]. In addition, the *pickpocket* gene is specifically expressed in the mdIV neurons and it is required for mechanical nociception [8]. Although Painless is expressed in all multidendritic neurons, only the class IV neurons have been found to be activated at high temperatures [6]. This latter finding suggests that tissue specific factors are likely to influence Painless activity.

With regard to mechanical nociception, the role of Painless is more poorly understood. The NEL responses to noxious mechanical stimulation have an increased threshold in *painless* mutant animals relative to wild type larvae which indicates an *in vivo* requirement for Painless in mechanical nociception responses [4]. However, *in vitro* studies on Painless expressed in a human embryonic kidney cell line failed to detect Painless dependent currents following hypo/hypertonic stimulation or direct touch with a glass pipette [3]. This failure to detect mechanical currents in Painless expressing cells may indicate that Painless is not directly mechanosensitive. An indirect role for Painless in mechanotransduction could occur if Painless functions downstream of another mechanotranduction signaling molecule in the mdIV neurons. Consistent with this possibility, the Degenerin/Epithelial Sodium Channel (DEG/ENaC) protein Pickpocket is required for mechanical nociception in *Drosophila* larvae but it is not required for thermal nociception [8]. In addition, Painless channel activity is strongly affected by intracellular Ca^2+^ which may provide a potential molecular mechanism for activation downstream of neuronal activity [3]. Thus, Painless may have a direct role in thermosensation but it may function downstream of Pickpocket in mechanical signaling pathways.

On the other hand, heterologous expression studies are difficult to interpret since mechanosensative channels may require specialized signaling components that may not be present in heterologous cells [9]. This may be particularly true with expression of an insect channel in mammalian cells since the required components in a mammalian cell, even if present, may not be capable of interacting with the channel. In the best-understood mechanotransduction system of *C. elegans*, the mechanotransduction complex relies on the pore forming DEG/ENaCs MEC-4 and MEC-10, extracellular proteins (MEC-1, MEC-5, and MEC-9) and additional intracellular components [10]. Similarly, in *Drosophila*, the extracellular protein NompA, is required for the connection between the mechanosensory neuronal sensory endings and cuticle in bristle mechanotransduction [11]. The identification of roles for extracellular proteins has led to a model of mechanosensation involving tethers that anchor the mechanosensitive channel or complex to intracellular and/or extracellular components that efficiently transmit force to the mechanosensitive channel.

Given the polymodal role of Painless in thermal nociception and in mechanical nociception we were interested in the possibility that particular domains of the Painless protein might play an important role in various aspects of signaling. Here, we report three naturally occurring RNA variants of *painless* that are predicted to encode Painless protein isoforms which vary in the length of the ankyrin repeat containing N-terminal domain. We utilize these naturally occurring variants as tools to investigate the functional properties of the ankyrin repeat domain of Painless through isoform specific rescue experiments *in vivo*. Our results indicate that the longest isoform, which contains the entire N-terminal ankyrin repeat domain, is sufficient to rescue thermal nociception of a *painless* mutant but does not rescue mechanical nociception. In addition, this long protein isoform shows efficient localization to dendrites and axons of the multidendritic neurons. In contrast, the shortest isoform, which essentially lacks an ankyrin repeat domain, is capable of rescuing mechanical nociception without rescuing thermal nociception. This short isoform does not efficiently localize to dendrites and is expressed at much lower levels in comparison to the long isoform. Combined, these results suggest that the polymodal functions of Painless may be explained by distinct protein variants that have modality specific properties. Furthermore, our results suggest that the N-terminal domain of Painless is required for thermal nociception, but we do not find evidence that it is required for mechanical nociception.

## Results

### Distinct isoforms of Painless vary in the length of their N-terminal domain

Three transcripts of *painless* are detectable by northern blot analysis [12]. To identify these different transcripts of *painless*, we performed 5′ Rapid Amplification of Complementary cDNA Ends (5′ RACE). Three 5′ RACE products were cloned and sequenced and determined to represent three distinct 5′ ends for *painless* transcripts. One of the RACE products was identical to the previously described 5′end of *painless* while the remaining two RACE products encoded novel 5′ ends. A search of the expressed sequence tag (EST) database identified 5′ EST sequences that exactly matched the 5′ ends of the *painless* RACE products. Only a single 3′EST sequence for *painless* transcripts is found in the database and this 3′end is identical to that of the canonical *painless* transcript. Combined, these data are consistent with the existence of three *painless* transcripts that differ in the 5′ sequences but which share a common 3′end. Conceptual translation of the novel transcripts predicted proteins that differed in the translational start site of their N-terminal domains. Based on the predicted molecular weights of the isoforms (103 kD, 72 kD and 60 kD), we refer to the predicted protein isoforms as Painless^p103^, Painless^p72^, and Painless^p60^.

**Figure 1:**
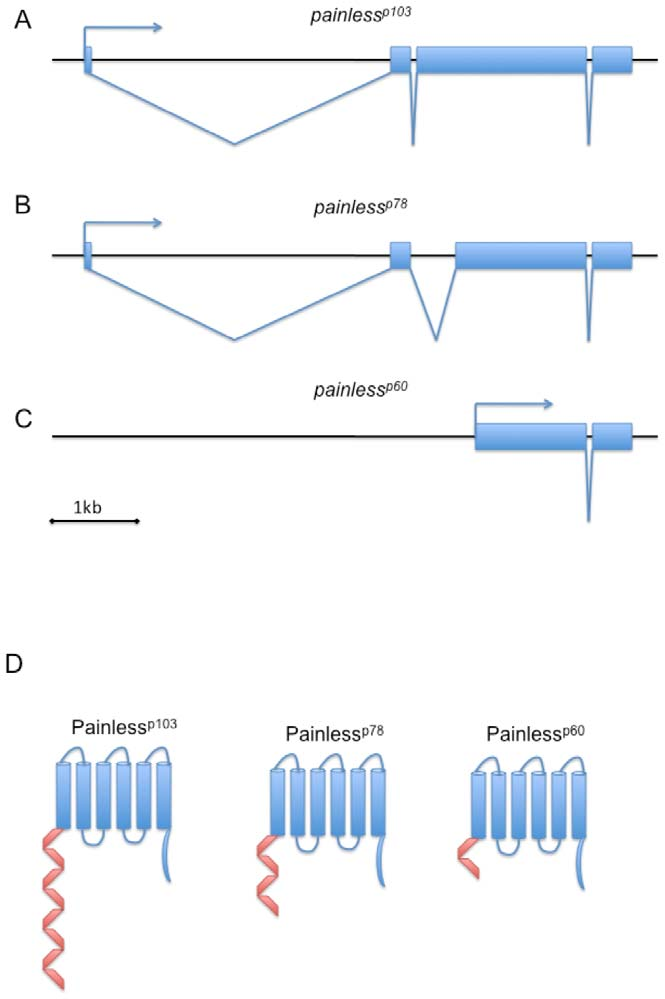
Schematic diagram of transcription units and proteins for the newly identified *painless* transcripts. (A.) The *painless^p103^* transcription unit. (B.) The *painless^p78^* transcript shares the first non-coding exon of *painless^p103^* but utilizes an alternative downstream splice acceptor. (C.) The *painless^p60^* transcript initiates from an alternate promoter that is downstream of the promoter for *painless^p103^* and *painless^p78^*. (D.) The predicted proteins for the three isoforms differ in the length of the n-terminal domain. Ankryin repeats are depicted as red.

The longest (canonical) isoform, Painless^p103^, is predicted to have 8 N-terminal ankyrin repeats, 6 transmembrane domains, and an intracellular carboxy-terminal domain (CTD) [4]. Note that four of the ankyrin repeats of Painless are somewhat degenerate in amino acid sequence, and are thus not detected by ankyrin repeat algorithms. The intracellular amino terminal domain (NTD) which contains the ankyrin repeats consists of the first 468 amino acids of Painless ^p103^. The transcript encoding the intermediate length isoform, Painless^p72^, is transcribed from the same promoter as the transcript encoding Painless^p103^, but it includes an alternately spliced second intron that results in the utilization of an alternate ATG start codon (Figure 1). The result of this splicing causes the NTD of Painless^p72^ to lack the first 285 amino acids of Painless^p103^. The shortest isoform, Painless^p60^, uses an alternate transcriptional start site that is downstream of the transcriptional start site of the other two transcripts (Figure 1). This structure causes the Painless^p60^ protein to lack the first 385 amino acids of Painless ^p103^. The resulting intracellular NTD of Painless^p60^ is a relatively short 83 amino acids. Interestingly, the alternate protein isoforms for Painless are similar in structure to N-terminal protein variants of other TRP channels. For example, there are two known protein isoforms of the *Drosophila* TRPA channel Pyrexia: Pyx-A and Pyx-B. As in the case of Painless^p60^, the Pyx-B protein is lacking the N-terminal ankyrin repeat containing domain while the Pyx-B protein resembles Painless^p103^ [13]. Similarly, the mammalian TRPV1 channel has a reported isoform that lacks ankyrin repeats at its N-terminus [14].

### Generation of transgenic flies expressing fluorescently tagged Painless^p103^ and Painless^p60^

**Figure 2:**
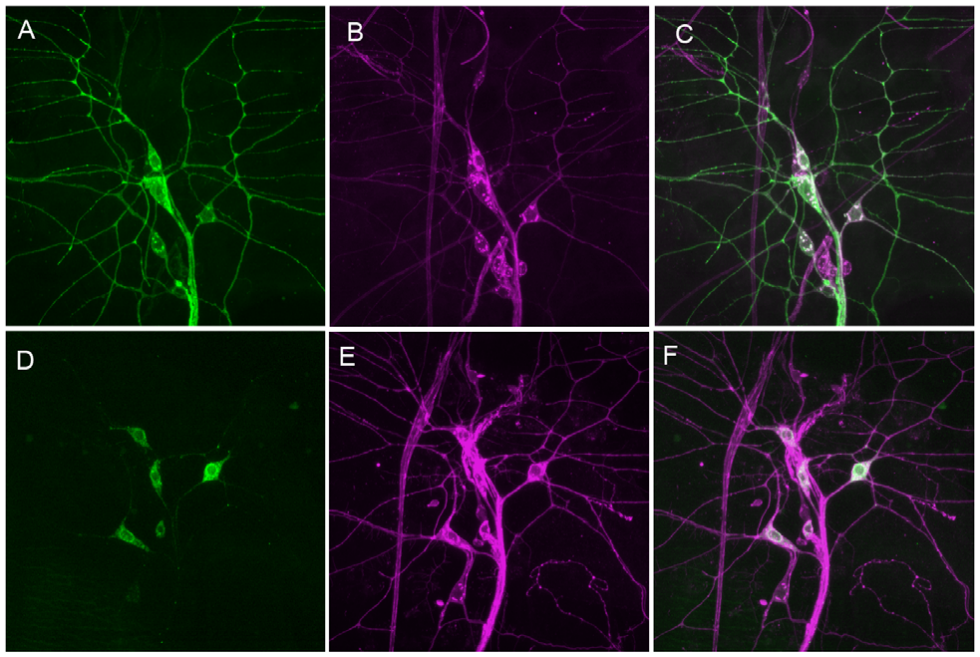
Distinct subcellular localization of Painless^p103^::VFP and Painless^p60^::VFP. A–F show the dorsal cluster of multidendritic neurons of larval abdominal segments immunostained with anti-GFP antibody (green) and anti-HRP (magenta). (A) Anti-GFP staining of *pain^Gal4^ UAS-Painless^p103^::VFP* larva. (B) Anti-HRP staining of *pain^Gal4^ UAS-Painless^p103^::VFP*. (C) Merge of A and B. (D) Anti-GFP staining of *pain^Gal4^ UAS-Painless^p60^::VFP* line 1. (E) Anti-HRP staining of *pain^Gal4^ UAS-Painless^p60^::VFP* line 1. (F) Merge of D and E. Note that the exposure times for acquisition of Painless^p60^::VFP signal was significantly longer than for Painless^p103^::VFP.

In order to test the functional roles of the Painless protein isoforms *in vivo*, we generated transgenic files to specifically express either the Painless^p103^ isoform or the Painless^p60^ isoform under control of the GAL4/UAS system. In order to estimate expression levels of the transgenes, an in-frame fusion of the Venus Fluorescent Protein (VFP) was added at the C-terminus of both constructs. We refer to these transgenes as *UAS-painless^p103^::VFP* and *UAS-painless^p60^::VFP*. To examine the localization of the VFP tagged Painless proteins, we crossed flies harboring *UAS-painless^p103^::VFP* or *UAS-painless^p60^::VFP* to the *painless^GAL4^* driver strain. In the case of *UAS- painless^p103^::VFP* the progeny of this cross showed robust VFP fluorescence throughout the dendrites, cell bodies, and axons of the *painless^GAL4^* expressing multidendritic sensory neurons (Figure 2A–C). This fluorescence was easily detectable in living animals using confocal microscopy (data not shown). The expression levels produced from this transgene exceeded that of the endogenous Painless protein as detected by increased immunostaining with anti-Painless antibodies relative to the wild type (data not shown). Indeed, wild type Painless protein is localized to discrete punctae [4], while the over-expressed VFP-Painless was present throughout the dendritic arbor. This wider distribution is likely a consequence of relatively high expression levels generated by the GAL4/UAS system.

In contrast, the VFP of UAS- painless^p60^::VFP was significantly less intense. The VFP fluorescence from *UAS- painless^p60^::VFP* was difficult to visualize in living larvae but was detectable by anti-GFP immunostaining of fixed preparations (Figure 2 C–D). Interestingly, the subcellular localization of Painless^p60^::VFP was distinct from that of Painless^p103^::VFP. Although Painless^p60^::VFP was easily detectable in the cell body the expression in dendrites and axons was limited to the most proximal regions (Figure 2 D–F). The subcellular localization and expression levels of the Painless^p60^::VFP was observed in multiple independent UAS insertion lines so it appears to be a property of the protein isoform as opposed to position effects that might limit expression levels of a particular UAS transgene insertion. These results suggest that the N-terminal domain of Painless results in enhanced stability of the Painless^p103^::VFP protein and more efficient localization to the dendrites and axons of multidendritic neurons relative to the Painless^p60^::VFP protein. Alternatively, the *painless^p60^* transcript may be unstable, or poorly translated, relative to the *painless^p103^* transcript.

### Pronounced thermal and mechanical nociception defects in the *pain^Gal4^*/*pain^NP7022^* allelic combination

In order to study the potentially distinct roles of the Painless^p103^::VFP and Painless^p60^::VFP isoforms, we performed genetic rescue experiments in *painless* expressing tissues. To achieve this, we took advantage of mutant alleles of *painless* that express the yeast transcription factor GAL4 in *painless* expressing cells. *pain^Gal4^* is one such mutant for *painless* which contains a GAL4 enhancer trap P-element insertion in the 5′ untranslated region (UTR) of the transcript encoding for Painless^p103^ and the shared 5′ UTR of the transcript encoding Painless^p72^. The *pain^Gal4^* allele shows GAL4 expression in multidendritic neurons, chordotonal neurons, and a subset of neurons in the CNS [4].

**Figure 3:**
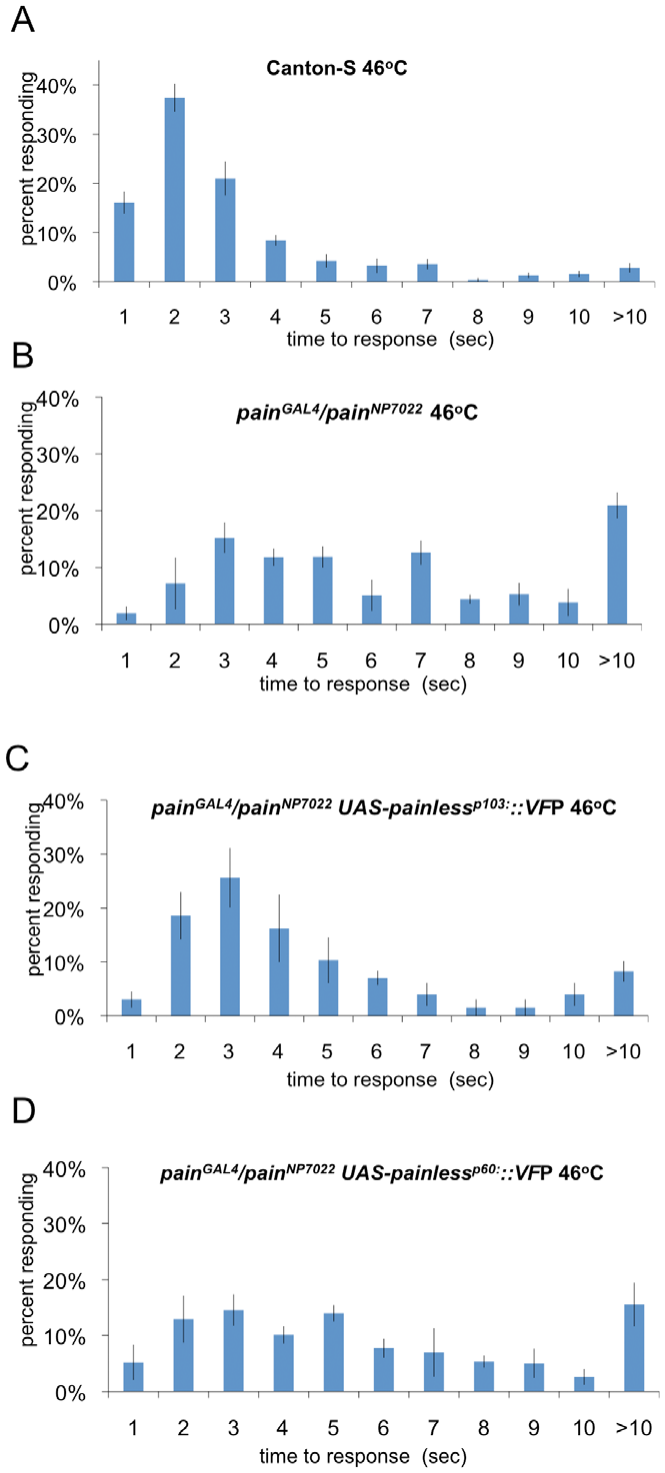
Isoform specific rescue of thermal nociception in *painless* mutant animals. (A) Nocifensive response latency of control (Canton-S) larvae, n = 235. (B) *pain^NP7022^*/*pain^Gal4^* larvae are defective in thermal nociception, n = 122 (compared to Canton-S(p<0.001). (C) Expression of *painless^p103^::*VFP in NP7022/pain-Gal4 partially rescues the thermal nociception phenotype (statistically different from both Canton-S (p<0.001 and NP7022/pain-Gal4 (p<0.001)) (D) Expression of *painless^p60^::*VFP does not rescue the thermal nociception phenotype of *pain^NP7022^*/*pain^Gal4^* n = 141 (statistically different from Canton-S(p<0.001) but not different from NP7022/pain-Gal4 (p>0.05)). The Wicoxon Rank Sum test was used for all statistical analysis.

**Figure 4:**
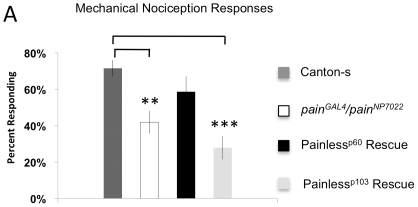
Isoform specific rescue of mechanical nociception phenotypes in *painless* mutant animals. The graph shows the percentage of animals that respond to 50 mN Von Frey stimulus with Nocifensive Escape Locomotion behavior. Canton-S n = 249, *pain^NP7022^*/*pain^Gal4^* n = 201, *pain^NP7022^*/*pain^Gal4^*; UAS-*painless^p60^::VFP* n = 343, *pain^NP7022^*/*pain^Gal4^* n = 201, *pain^NP7022^*/*pain^Gal4^*; UAS-*painless^p103^::VFP* n = 71. (One way ANOVA p<0.0001, pair-wise comparisons to Canton-S were performed with the Dunnett post-hoc multivariate t-distribution, **indicates p<0.01 and *** indicates p<0.001 relative to Canton-S).

To further dissect the role of distinct molecular features Painless protein isoforms in either mechanical or thermal nociception, we used expression of GAL4 in the *pain^Gal4^* mutant allele in combination with the *pain^NP7022^* allele. Mutant animals with the *pain^Gal4^*/*pain^NP7022^* genotype showed pronounced thermal nociception and mechanical nociception defective phenotypes. In wild type larvae gently touched with a probe heated to a noxious temperature (46°C), nocifensive escape behavior was rapidly triggered (Figure 3A). In contrast, *pain^Gal4^*/*pain^NP7022^* larvae showed a response that is typical for other mutant alleles of *painless* (Figure 3B). In mechanical nociception tests, wild type larvae stimulated with a 50 mN Von-Frey fiber showed nocifensive responses in 70% of trials whereas *pain^Gal4^*/*pain^NP7022^* trans-heterozygous larvae only responded approximately 40% of the time (Figure 4). Thus, *pain^Gal4^*/*pain^NP7022^* trans-heterozygotes exhibited robust thermal and mechanical nociception defective phenotypes.

### Modality specific isoforms of Painless

We next tested whether expression of the Painless^p103^::VFP or the Painless^p60^::VFP proteins would be sufficient to rescue the polymodal aspects of the painless nociceptive phenotype. We hypothesized that the Painless^p103^ isoform would functionally rescue mechanical nociception because of the predicted role the N-terminal domain ankyrin repeats in the gating spring model for mechanosensation. In contrast, the Painless^p60^::VFP protein might rescue thermal nociception but fail to rescue mechanical nociception.

The results were contrary to these expectations as expression of the Painless^p103^::VFP in the *pain^Gal4^*/*pain^NP7022^* mutant background (*pain^Gal4^*/*pain^NP7022^*; UAS-Painless^p103^::VFP/+) showed a nearly complete rescue of nociception responses to a 46°C thermal stimulus (Figure 3A-C). In contrast to the rescue experiments using UAS-Painless^p103^::VFP trangenes, the *pain^Gal4^*/*pain^NP7022^*; UAS Painless^p60^::VFP/+ animals did not show rescue of the mutant responses to thermal nociception stimuli (Figure 3D).

The failure of Painless^p60^::VFP to rescue thermal nociception might have been due its lower expression levels in the sensory neurons or it might indicate that Painless^p60^::VFP encodes a non-functional channel. Thus to further test the functional properties of Painless^p60^::VFP we tested if it could rescue defective mechanical nociception behaviors of *pain^Gal4^*/*pain^NP7022^* mutants. Expression of this transgene in the mutant background improved the mechanical nociception responses of the mutant animals (Figure 4) such that they were no longer different from wild type levels. This result suggested that the Painless^p60^::VFP transgene was functional, and that the expression level of this transgene was sufficient to rescue mechanical nociception, but not thermal nociception *painless* mutant phenotypes.

Most surprisingly, the Painless^p103^::VFP protein, which was expressed at significantly higher levels than Painless^p60^::VFP, was unable to rescue mechanical nociception (Figure 4). If anything, expression of Painless^p103^::VFP enhanced mechanical nociception defects of the *pain^Gal4^*/*pain^NP7022^* mutant larvae. These results do not conform to predictions of the gating spring model for the ankyrin repeat domain. Our results suggest that the ankyrin repeat domain of Painless^p103^ is important for thermal signaling and not for mechanical signaling. The Painless^p60^ isoform, lacking ankyrin repeats shows complimentary functions, rescuing mechanical signaling without rescue of thermal signaling.

## Discussion

We have found three isoforms of Painless that vary in the length of the ankyrin repeat containing N-terminal domain with the longest isoform containing the full N-terminal domain and the shortest isoform, containing only a small portion of the N-terminal domain. The Painless^p103^ isoform is capable of rescuing the thermal nociception phenotype of mutant animals in the absence of functional rescue for mechanical nociception phenotypes. The Painless^p60^ short isoform is capable rescuing mechanical nociception in the absence of functional rescue for thermal nociception. These findings do not support a previously proposed hypothesis in which ankyrin repeats serve as a compliant gating spring in mechanosensing TRP channels.

Our results suggest the possibility that distinct isoforms of Painless are dedicated to specific sensory modalities which require Painless function. The Painless^p103^ isoform may primarily be required for thermal nociception whereas the Painless^p60^ isoform may be specifically involved in mechanical nociception. Interestingly, this finding suggests that the N-terminal domain of Painless may have an important function in temperature sensing rather than in mechanotransduction. Note that our data do not exclude the possibility that more than one isoform of Painless may be present in functional channels *in vivo*. This caveat is necessary to consider because the *pain^Gal4^*/*pain^NP7022^* mutant larvae are not null for the *painless* locus. Residual expression of the different isoforms may allow for the formation of heteromeric channels in our rescue experiments despite the fact that the rescue transgenes are designed to express a single isoform. To determine definitively whether the distinct isoforms are genuinely sufficient for modality specific rescue, the experiments described here must be repeated in a DNA null mutant for *painless*. Efforts to generate a null allele for *painless* in our laboratory will make this ideal experiment possible in the near future. In addition, a *painless* null allele would allow for tissue specific rescue experiments in the nociceptor neurons. Interpretation of the results of this study must be tempered by the caveat that pain-GAL4 is expressed in cells that are not nociceptive in addition to the nociceptors themselves.

In the case of mechanosensitive TRP channels, it has been proposed that the NTD may play an important role in transmitting cytoskeletal force to the channels [15]. This theory was developed largely based upon the unusually large number (twenty-nine) of ankyrin repeats found at the N-terminus of Nomp-C [15]. The Nomp-C channel is a TRP channel that was first identified in a screen for uncoordinated mutants that had defects in mechanically induced currents in *Drosophila* bristle neurons [16]. Interestingly, Nomp-C is also required for hearing in both Drosophila and Zebrafish [17], [18]. In addition, a conserved role for Nomp-C in mechanotransduction has been found in *C. elegans* [19]. The ankyrin repeats of Nomp-C have been hypothesized to function as a flexible spring that can transmit force to the mechanosensitive channel possibly while being anchored to a cellular component such as the cytoskeleton [15]. Consistent with this gating spring hypothesis, evidence has also shown that the ankyrin repeats of ankyrin-R indeed behave as a Hookean molecular spring [20]. Widespread interest in this theory stems from the fact that evidence suggests that a flexible spring element may be involved in gating of the elusive mechanosensitive ion channel in hair cells of the inner ear. It was originally proposed that the extracellular tip links near the tips of stereocilia might be the gating spring. However, the identification of cadherin-23 [21], [22], and protocadherin-15 as components of tip links [23] argued against tips-links serving as the gating spring because molecular simulations predict that cadherin molecules are stiffer than the “gating spring” in hair cells [24]. These simulations provided further support to the idea that the compliant spring element in hair cells might exist intracellularly [25].

A potential role for the NTD in thermoTRP heat sensing has also been previously suggested. In the case of TRPV4, deletion of ankyrin repeats abolishes heat activated currents [26] but not its response to hypotonic stimulation [27]. Even so, a role for the NTD in temperature sensing may not be generally applicable to all thermoTRP channels. The two isoforms of Pyrexia, a thermosensitive Drosophila TRPA channel differ in the presence of the N-terminal domain and yet both are thermosensitive [13]. This suggests that the N-terminal domain of Pyrexia may not be required for heat activation. Still other evidence implicates the CTD of TRPV1 and TRPM8 in thermosensing. Deletion of the CTD alters TRPV1's temperature sensitivity and domain swapping of the TRPM8 CTD with the TRPV1 CTD causes a switch of function in thermosensitivity [28], [29]. In addition, residues surrounding the pore have been implicated in allosteric modulation of temperature sensing of TRPV3 and TRPV1.

Interestingly, recent results from our laboratory [30] and others [31] support the idea that the amino terminal domains of TRPA1 channels play a role in heat sensing. Alternative splicing of *Drosophila TrpA1 (dTrpA1)* generates transcripts that encode either heat sensitive (dTRPA1-A, dTRPA1-D) or heat insensitive isoforms of dTRPA1 (dTRPA1-B, dTRPA1-C) [30]. TRP Ankryin Caps (TACs) vary between the various dTRPA1 isoforms and the TACs determine the heat sensing properties. The dTRPA1-C heat insensitive isoform was found to be required for thermal nociception but not mechanical nociception. It is possible that the mechanical nociception function of *dTrpA1* could rely on an as yet unidentified isoform for this channel that lacks ankyrin repeats as we have now found for *painless*.

In addition, chimeric channels between heat sensitive snake or *Drosophila* TRPA1 channels made with the heat insensitive human TRPA1 channel, support the idea that heat sensor domains reside within the ankyrin repeat region [31]. Our *in vivo* analyses of *painless* are in definite support of this idea.

Although the Painless NTD is apparently not required for mechanical nociception it would be premature to extrapolate from this finding to other TRP channels involved in mechanotransduction. Additional experiments will be necessary to test whether or not Painless or other TRPA channels encode pore-forming subunits of mechanosensory channels. As mentioned above, Painless may function downstream of another mechanosensory such as Pickpocket. Interestingly, our results suggest that if Painless does function downstream of Pickpocket, then the method of activation for Painless is unlikely to be dependent on Ca^2+^ influx, since the putative EF hand of the Painless NTD in absent in the Painless^p60^ isoform.

Our results also indicate that the Painless ankyrin repeats play an important role in the sub-cellular localization and the expression level of Painless isoforms. The Painless^p60^::VFP transgene was expressed at relatively low levels *in vivo* and its subcellular localization was restricted to proximal dendrites, the cell soma, and proximal axons. In contrast, the Painless^p103^::VFP isoform was expressed robustly and throughout the multidendritic neurons including the sensory dendrites. These findings suggest the possibility that Painless^p103^::VFP and Painless^p60^::VFP proteins function in distinct subcellular compartments for thermal and mechanical nociception. Thermal nociception signaling by Painless ^p103^ may occur in the more distal sensory dendrites whereas the mechanical nociception function for Painless may reside in proximal regions of dendrites, likely in amplification of upstream mechanosensory transduction signals initiated by the DEG/ENaC Pickpocket.

## Materials and Methods

### RNA Ligase Mediated Rapid Amplification of cDNA Ends (RLM-RACE)

The 5′ends of *painless* transcripts were isolated by 5′ RACE using the FirstChoice (RLM-RACE kit (RNA Ligase Mediated Rapid Amplification of cDNA Ends, Ambion Inc.) according to the instructions of the manufacturer. RNA was isolated from a mixed population of first and second instar *Drosophila w^1118^* larvae using Trizol reagent (Invitrogen/Life Technologies) and treated with DNAse-I (DNA-free Kit (Ambion Inc.). The 5′ phosphate from any uncapped RNA in the preparation was then removed by treatment with Calf Intestine Alkaline Phosphatase (CIP). The CIP reaction was terminated by phenol-chloroform extraction, and the RNA was precipitated with isopropanol. The 5′ cap was removed from the mRNA by treatment with Tobacco Alkaline Pyrophosphatase (TAP) enzyme, leaving monophosphated 5′ ends. A 45-base RNA adaptor from the FirstChoice (RLM-RACE kit was then ligated to these newly exposed monophosphated 5′ ends with T4 RNA Ligase.

First strand cDNA was generated using random decamer primers and M-MLV Reverse Transcriptase. Nested PCR was performed to amplify *painless* 5′ ends, using forty cycles and 5 min extension times for both inner and outer reactions. The forward primers were supplied by the RACE kit (5′ Race outer and inner forward primers) and are complimentary to sequences present in the ligated 5′ adaptor. The outer reaction *painless* specific reverse primer 5′-GGATGGTAAATACGGCTAAGAC-3′ and the inner reaction painless-specific reverse primer 5′-TTCGTGGAACTTGAGGAGGCGTG-3′ were used. PCR products were examined by agarose-TAE gel electrophoresis. The products were gel-purified, cloned into pCR-XL-TOPO cloning vector (Invitrogen/Life Technologies), and introduced into electrocompetent *E.coli* of the TOP10 strain (Invitrogen/Life Technologies). DNA from Kanamycin-resistant colonies was digested with EcoRI restriction endonuclease to release the cloned inserts, and the relative molecular weight of the inserts was determined by gel electrophoresis. Clones containing inserts with unique molecular weights were selected for sequencing. The inserts of clones were sequenced using M13 F and M13R against the TOPO XL vector.

### Fly Strains

To generate the UAS-*painless^p103^::YFP* and UAS-*painless^p60^::YFP* plasmids the open reading frames were amplified from BACR08I14 using the forward primer for *painless^p103^* 5′ CAC CAT GGA CTT TAA CAA CTG C ′3 or the forward primer for *painless ^p60^* 5′ CACCATG GAT ATC AAC TCG AGA CCA 3′. The reverse primer 5′ CCG GTC CTG GAC CAG CT 3′ was used for both constructs. The PCR products for the respective gene products were then cloned in the pENTR-D vector (Invitrogen) for use with the *Drosophila* Gateway Cloning System. The resulting *painless ^p60^* and *painless^p103^* ENTR vectors were then used as substrates for clonase mediated recombination with the pTWV destination vector. Fully sequenced inserts from this reaction were found to contain wild type *painless* sequences and were used to transform the *w^1118^ Drosophila* strain via P-element mediated transformation. Inserts from transformed flies were mapped to a chromosome and the expression levels of the VFP transgenes were determined by crossing to the *painless^GAL4^* driver strain. For behavioral experiments inserts on the third chromosome were used. Flies with the genotype *w;painless^GAL4^*, *w; painless^GAL4^*; UAS-*painless^p103^::YFP/*K87 (T(2∶3) SM6a TM6b *Cy Tb*), or *painless^GAL4^*; UAS-*painless^p60^::YFP/*K87 were crossed to *pain^NP7022^/*K87 and *Tb^+^* progeny were selected and tested for nociception behavioral responses as described previously.

### Immunostaining and confocal microscopy

For visualization of the painless isoforms, the *pain^GAL4^* driver was crossed *to UAS-painless^P103^::VFP* (venus fluorescent protein) or *UAS-painless^P60^::VFP*. Trans-heterozygous larvae were dissected and filleted in Ca^2+^ free HL3 saline (70 mM NaCl, 5 mM KCl, 20 mM MgCl_2_, 10 mM NaHCO_3_, 5 mM trehalose, 115 mM sucrose, and 5 mM HEPES [pH 7.2]) followed by fixation for 1 hour in 4% paraformaldehyde. Primary mouse anti-GFP (1∶1000) and anti-mouse Alexa Fluor 488 (Molecular probes, 1∶1000) secondary were used to detect VFP. Neurons were counterstained with rabbit anti-HRP (1∶500, anti-horseradish peroxidase) and the secondary anti-rabbit Alexa Fluor 568 (Molecular probes, 1∶1000). Images (1024×1024) were taken with a Zeiss LSM 5 Live Confocal system using a 40×1.3 N/A oil immersion lens. The two channels were collected separately in multi-track mode (anti-GFP: excitation 488 nm, emission 500–525 nm) (anti-HRP excitation 532 nm, emission 560–675). The confocal micrographs are presented maximum intensity projections of confocal Z-stacks.
